# Fine *De Novo* Sequencing of a Fungal Genome Using only SOLiD Short Read Data: Verification on *Aspergillus oryzae* RIB40

**DOI:** 10.1371/journal.pone.0063673

**Published:** 2013-05-07

**Authors:** Myco Umemura, Yoshinori Koyama, Itaru Takeda, Hiroko Hagiwara, Tsutomu Ikegami, Hideaki Koike, Masayuki Machida

**Affiliations:** 1 Bioproduction Research Institute, National Institute of Advanced Industrial Science and Technology (AIST), Sapporo, Hokkaido, Japan; 2 Bioproduction Research Institute, National Institute of Advanced Industrial Science and Technology (AIST), Tsukuba, Ibaraki, Japan; 3 Department of Biotechnology and Life Science, Tokyo University of Agriculture and Technology, Koganei, Tokyo, Japan; 4 Information Technology Research Institute, National Institute of Advanced Industrial Science and Technology (AIST), Tsukuba, Japan; Technical University of Denmark, Denmark

## Abstract

The development of next-generation sequencing (NGS) technologies has dramatically increased the throughput, speed, and efficiency of genome sequencing. The short read data generated from NGS platforms, such as SOLiD and Illumina, are quite useful for mapping analysis. However, the SOLiD read data with lengths of <60 bp have been considered to be too short for *de novo* genome sequencing. Here, to investigate whether *de novo* sequencing of fungal genomes is possible using only SOLiD short read sequence data, we performed *de novo* assembly of the *Aspergillus oryzae* RIB40 genome using only SOLiD read data of 50 bp generated from mate-paired libraries with 2.8- or 1.9-kb insert sizes. The assembled scaffolds showed an N50 value of 1.6 Mb, a 22-fold increase than those obtained using only SOLiD short read in other published reports. In addition, almost 99% of the reference genome was accurately aligned by the assembled scaffold fragments in long lengths. The sequences of secondary metabolite biosynthetic genes and clusters, whose products are of considerable interest in fungal studies due to their potential medicinal, agricultural, and cosmetic properties, were also highly reconstructed in the assembled scaffolds. Based on these findings, we concluded that *de novo* genome sequencing using only SOLiD short reads is feasible and practical for molecular biological study of fungi. We also investigated the effect of filtering low quality data, library insert size, and k-mer size on the assembly performance, and recommend for the assembly use of mild filtered read data where the N50 was not so degraded and the library has an insert size of ∼2.0 kb, and k-mer size 33.

## Introduction

Whole-genome sequencing is an invaluable tool in evolutionary and functional studies of biological systems. The development of next-generation sequencing (NGS) technologies, such as the SOLiD (Life Technologies), Solexa and Genome Analyzer (Illumina), and 454 GS FLX (Roche) systems, has increased the throughput and reduced the cost of sequencing by several orders of magnitude [1]. To date, the whole genomes of several viral [2], [3], bacterial [4]–[8], and fungal species [2], [9]–[12] have been newly sequenced (*de novo* sequencing) by combining two or more NGS platforms, such as 454 and Solexa, which generate sequence reads of 250∼800 bp and ∼100 bp, respectively. *De novo* sequencing using only a single NGS platform such as Illumina and SOLiD can further reduce costs and time. Among several reports of such *de novo* sequencing from bacterial to mammalian genomes [4]–[8], [10], [12]–[15], the most successful result was shown for human and mouse genomes with the Illumina platform by using various libraries, in which the scaffold sizes (N50 size  = 11.5 and 7.2 Mb for human and mouse genomes, respectively) approach those obtained with traditional capillary-based sequencing [14]. As compared to the Illumina reads, few successful reports of *de novo* sequencing, especially of eukaryotes, are found in the case of the SOLiD read (∼60 bp). Fine *de novo* sequencing using only SOLiD reads was reported for the genome of a bacterium *Corynebacterium pseudotuberculosis* [13], but the N50 length is 76.9 kb even using two assembly methods in combination. The SOLiD short sequence read is generally suspected to be too short for *de novo* sequencing especially when a genome includes introns and long repetitive sequences. In addition, there are only a few assemblers that can deal with the “color-space” format of SOLiD read data to date.

Filamentous fungi produce a wide range of secondary metabolites, such as penicillin, cyclosporin, and lovastatin, with useful medicinal, agricultural, and cosmetic properties [16]–[18]. As the genome sequencing of fungi can provide information related to secondary metabolite biosynthesis (SMB) genes, which often contain characteristic sequence motifs [19], [20], the *de novo* sequencing of fungal isolates is anticipated to facilitate the identification of novel SMB genes. Fungal genome sequences have characteristics that make them difficult to be sequenced in comparison to bacterial ones; abundant repeat sequences especially in SMB genes, and AT-rich centromere sequences. Since SMB genes are clustered in general, sequence information longer than 50 kb is required for their identification. For example, the SMB gene cluster of aflatoxin, which is one of the most carcinogenic fungal secondary metabolites identified to date, occupies a region of ∼77 kb in the *Aspergillus flavus* genome [21]. Recently, we successfully identified the SMB gene cluster of kojic acid (KA) [22], which is used in cosmetics as a skin-whitening agent, using the genome sequence data of *Aspergillus oryzae* [23], including the genomic location of two genes involved in KA synthesis. *De novo* sequencing of fungal genomes using only the SOLiD platform is expected to facilitate the rapid and efficient identification of novel SMB gene clusters, when the assembled sequences are sufficiently long and accurate for the identification.

To investigate whether the SOLiD short sequence read is useful for *de novo* sequencing of fungal genomes, we conducted a series of *de novo* assemblies of the filamentous fungus *A. oryzae* RIB40 genome using only SOLiD reads of 50 bp in color-space obtained from a single sequencing run. The 37-Mb genome of RIB40 has been sequenced by the method of Sanger and was found to contain several SMB gene clusters, even though this strain rarely produces secondary metabolites [23]. Thus, we validated the results of our *de novo* assembly approach in terms of the size distribution of the assembled scaffolds, the reconstruction of the genome sequence including SMB gene clusters, and the sequence accuracy. In addition, we specifically evaluated three factors to assess the performance of the *de novo* genome assembly; (1) quality of the read data, (2) insert size of a mate-paired library, and (3) k-mer size used in the assembly program, by changing the degree of data filtering, the library insert size, and k-mer sizes.

## Materials and Methods

### Strain and medium

The fungal strain used in this study, *A. oryzae* RIB40, was obtained from the National Research Institute of Brewing, Japan (http://www.nrib.go.jp/ken/asp/strain.html). For DNA isolation, strain RIB40 was grown in liquid YPD (Yeast extract, Peptone, Dextrose) medium (Difco) at 30°C for 2 days.

### Whole genome sequencing

The genomic DNA was isolated from RIB40 as described previously [24]. We constructed two mate-paired libraries, lib2.8 and lib1.9, which were derived from sheared genomic DNA fragments of sizes 2.8 and 1.9 kb, respectively, using the SOLiD Long Mate-Paired Library Construction kit (Life Technologies, USA) and 50 μg RIB40 genomic DNA. Whole-genome sequencing using the lib2.8 or lib1.9 libraries was performed using the SOLiD 5500xl system (ABI). The mate-paired sequencing of lib2.8 and lib1.9 yielded 112347006 and 50526687 pairs of F3 and R3 reads, respectively, with lengths of 50 bp. The two sets of short read data are available at DDBJ Sequence Read Archive (http://trace.ddbj.nig.ac.jp/dra/index_e.shtml; accession number: DRA000909).

### Genome assembly

**Figure 1:**
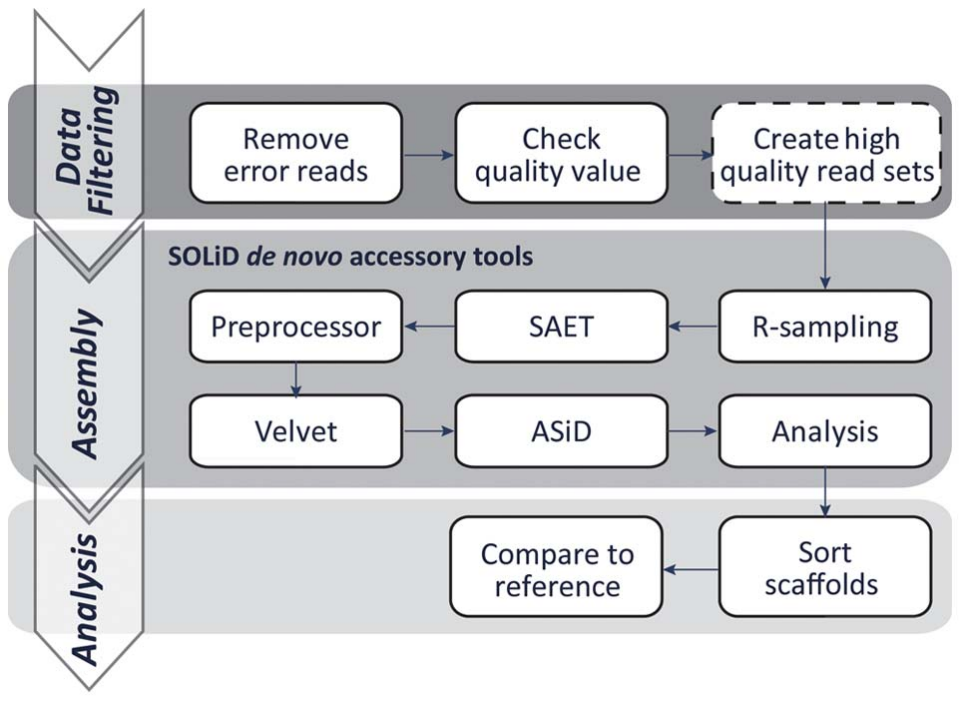
Overview of our *de novo* genome assembly pipeline. The assembly block is performed using SOLiD *De Novo* Accessory Tools 2.0 developed by Life Technologies. The data filtering and analysis blocks are written in shell, Ruby, or Perl languages.

Prior to genome assembly, an XSQ file for the F3 and R3 reads generated by the SOLiD platform was converted into a color read sequence file (csfasta) and quality file (.qual) using a shell script downloaded from the ABI website (convertFromXSQ.sh; http://www.lifetechnologies.com/us/en/home/technical-resources/software-downloads/xsq-software.html). To investigate the effect of data filtering on the assembly performance, we prepared three and two read sets from the lib2.8 and lib1.9 libraries, respectively, by changing degree of data filtering according to quality values (QV); unfiltered data (lib2.8.nofilter.k31), data excluding reads containing undetermined base(s) (designated by “.”; lib2.8.nodot.k31 and lib1.9.nodot.k31), and data in which all bases had QVs of >10 or 90% base-level accuracy (lib2.8.qv10.k31 and lib1.9.qv10.k31, Table 1). The depth of coverage of each read set on the reference genomic sequence of RIB40 (∼37 Mb, DDBJ: AP007150-AP007177; GenBank: AP007150-AP007177) is summarized in Table 2. After filtration, mate-paired reads were subjected to *de novo* assembly using SOLiD *De Novo* Accessory Tools 2.0 (Life Technologies), which includes the r-sampling, SAET, Velvet [25] (upgraded to version 1.0.15), and ASiD programs (Fig. 1). We modified ASiD to accelerate the gap-filtering process by eliminating the waiting time during parallel processing. We manually set the parameter for k-mer size (-hsize) to 31 or others, as described below, and used default values for other parameters (Table S1). It should be noted that scaffolds yielded by the SOLiD *De Novo* Accessory Tools consist of high quality nucleotide sequences without continuous undefined nucleotides (N), because continuous N is separated by the Analysis program. The assembly results from lib1.9.nodot.k31 and lib1.9.qv10.k31 were also used to examine the effect of mate-paired library insert sizes on the assembly performance. For both read sets of lib2.8.qv10 and lib1.9.qv10, we performed the assemblies with different k-mer sizes from 25 to 35 (restricted to odd integers), as k-mer size is a crucial parameter of the Velvet program. All of the assemblies are listed in Table 1. Assemblies were executed on a DELL precision T7500 desktop computer (CPU, Xeon E5620×2; Memory, 96 GB; Harddisk, 2TB×5; OS, Ubuntu Linux 10.04). An overview of our *de novo* assembly process is illustrated in Fig. 1. The pipeline built by YK will be provided on a request.

**Table 1:** Libraries, data filtering, and k-mer size used for the series of *de novo* assemblies.

Library	Mean size (bp)	SD^a^	Name	Quality value for filtering	*k*-mer
lib2.8	2764	500	lib2.8.nofilter.k31	none	31
			lib2.8.nodot.k31	no dot	31
			lib2.8.qv10.k31	10^b^	31
			lib2.8.qv10.k25	10^b^	25
			lib2.8.qv10.k27	10^b^	27
			lib2.8.qv10.k29	10^b^	29
			lib2.8.qv10.k33	10^b^	33
			lib2.8.qv10.k35	10^b^	35
lib1.9	1875	400	lib1.9.nodot.k31	no dot	31
			lib1.9.qv10.k31	10^b^	31
			lib1.9.qv10.k25	10^b^	25
			lib1.9.qv10.k27	10^b^	27
			lib1.9.qv10.k29	10^b^	29
			lib1.9.qv10.k33	10^b^	33
			lib1.9.qv10.k35	10^b^	35

a Standard deviation of the library insert size.

b Meaning all bases have QVs of >10 or 90% base-level accuracy.

**Table 2:** Performance of the *de novo* assemblies.

	Reads	Scaffolds						Contigs	
Name	Depth (×)^a^	Number (>95 bp)	Number (>500 bp)	N50 (kb)	Max (kb)	Coverage (%)^b^	R50 (bp)^c^	Number (>95 bp)	N50 (kb)
lib2.8.nofilter.k31	338	1184	317	1677	3363	98.87	29321	5332	16
lib2.8.nodot.k31	334	1147	303	1361	3361	98.85	29720	5265	16
lib2.8.qv10.k31	228	2751	465	983	3580	98.88	26708	7763	13
lib2.8.qv10.k25	228	4182	1042	1379	3334	99.10	21097	9448	13
lib2.8.qv10.k27	228	3790	858	1344	3288	99.15	23586	8960	13
lib2.8.qv10.k29	228	3220	648	1538	3328	98.45	25261	8321	13
lib2.8.qv10.k33	228	2260	331	1230	3270	99.21	27340	7179	14
lib2.8.qv10.k35	228	1774	194	1364	4240	99.15	27327	6702	13
lib1.9.nodot.k31	148	2456	426	913	2012	98.87	28417	7153	15
lib1.9.qv10.k31	117	3050	410	876	2032	99.05	29523	8254	13
lib1.9.qv10.k25	117	4468	773	775	1747	99.34	20412	10278	13
lib1.9.qv10.k27	117	4109	685	843	1983	98.96	22828	9704	13
lib1.9.qv10.k29	117	3601	538	876	1794	98.78	25830	9066	13
lib1.9.qv10.k33	117	2465	259	848	2013	98.94	29978	7545	13
lib1.9.qv10.k35	117	1828	173	820	2696	98.48	30813	6893	13

a Values after r-sampling; estimated based on the genome size of RIB40 (37 Mb).

b Estimated from scaffold alignments on the reference sequence by LAST.

c N50 values of scaffold fragments aligned on the reference sequence by LAST.

### Assessment of assembled scaffolds

We assessed the genome assembly performance using three criteria; size distribution of assembled scaffolds, degree of genome reconstruction, and sequence accuracy. Size distribution of assembled scaffolds were mainly estimated from the number and N50, which is defined as the length N for which 50% of all bases in the scaffolds are in a scaffold of length L < N. The maximum size and cumulative length of the assembled scaffolds was also considered. These results were generated by SOLiD *De Novo* Accessory Tools. To estimate the degree of genome reconstruction, we performed an alignment of the assembled scaffolds with the reference genome sequence by the LAST program [26]–[28], and used all pairs of nucleotide sequences having alignment scores of >40, which is a criterion for significant homology, for analysis of the genome coverage and misarrangement. Misjoins, deletions, insertions, and inversions of >500 bp in the reference nucleotide sequence were counted as misarrangements. We introduced a new statistic, R50, which is N50 for sequence fragments of the reference genome covered by highly accurate sequences of assembled scaffolds (having alignment score of >40 by LAST). In R50, the total bases of the fragments are supposed to be the size of the reference genome, like the NG50 statistic defined by Earl et al. [29]. To estimate sequence accuracy, nucleotide gaps (insertions and deletions) in the pairs of assembled scaffolds and genome sequence were counted.

To assess reconstruction of gene-coding regions, the nucleotide sequence of all RIB40 genes (including introns) was subjected to Blastn searches [30], [31] against the nucleotide sequences of assembled scaffolds (>95 bp), and the percentages of segment pairs having e-values of <1E-100 (high-scoring segment pairs, HSPs) and identical bases in the gene region were evaluated. For each gene, only the highest-scoring sequence was used for the evaluation. We also summarized the analysis results for polyketide synthase (PKS) and nonribosomal peptide synthetase (NRPS) genes in *A. oryzae* RIB40 when the gene annotation included “polyketide” and “non-ribosomal”, respectively (Table S2). For examining the gene continuity in the assembled scaffolds, two gene clusters of *A. oryzae*, AO090026000008−AO090026000036 (29 genes, ∼73 kb) and AO090001000018−AO090001000055 (38 genes, ∼75 kb), which were assigned as SMB gene clusters based on homology to the AFLA_139100-AFLA_139440 (aflatoxin biosynthesis) and AFLA_064330-AFLA_064650 (hypothetical gliotoxin biosynthesis) gene clusters, respectively, of *A. flavus*, were used.

As an additional analysis, the total coverage of reference sequence by the short reads was evaluated from mapping results using SHRiMP version 1.4 [32]. The open source program, MUMmer version 3.0 [33], was used to draw the dot plot of the assembled scaffolds in assembly lib2.8.nofilter.k31 over the reference genome sequence. All analyses were performed using in-house codes written in Perl language on the same desktop computer used for the genome assembly.

## Results

### Performance of our de novo assembly process

**Figure 2:**
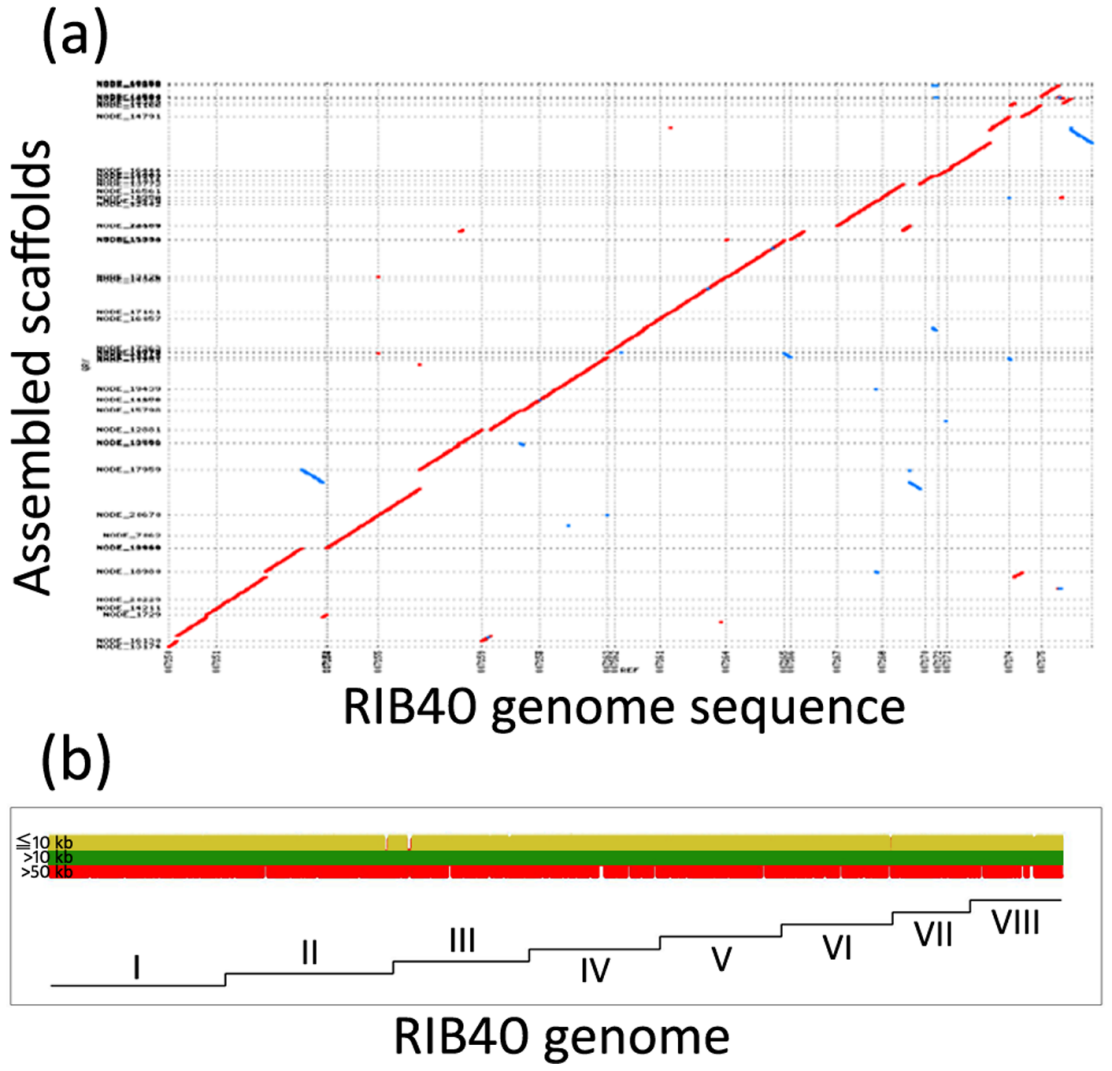
Coverage of the reference genome sequence by the assembled scaffolds. (a) Dot-plot alignments of assembled scaffolds vs the reference genome sequence of *Aspergillus oryzae* RIB40. (b) Reference genome sequences aligned by assembled scaffold fragments with lengths of ≤10 kb (yellow), >10 kb (green), and >50 kb (red). The Roman numerals I-VIII indicate the chromosome index of the RIB40 genome.

To determine the feasibility of *de novo* sequencing the RIB40 genome using only SOLiD short reads, performance of the genome assembly was evaluated based on the N50 values, maximum size, and total coverage of the reference sequence by the assembled sequences (Table 2). The assemblies were named by including the library number (lib2.8 or lib1.9), data filtering (nofilter, nodot, or qv10), and k-mer size (k25-k35, restricted to odd integers). The assembly lib2.8.nofilter.k31, which used unfiltered read data, was treated as the standard assembly. The standard assembly had an N50 of 1.7 Mb and a maximum scaffold size of 3.4 Mb without continuous undefined nucleotides. The coverage of the reference sequence by the assembled scaffolds reached 98.87% (Table 2). As shown in Figure 2, most regions of the reference sequence were covered by the assembled scaffolds that consisted of >10-kb fragments.

### Reconstruction of gene regions and SMB gene clusters

**Figure 3:**
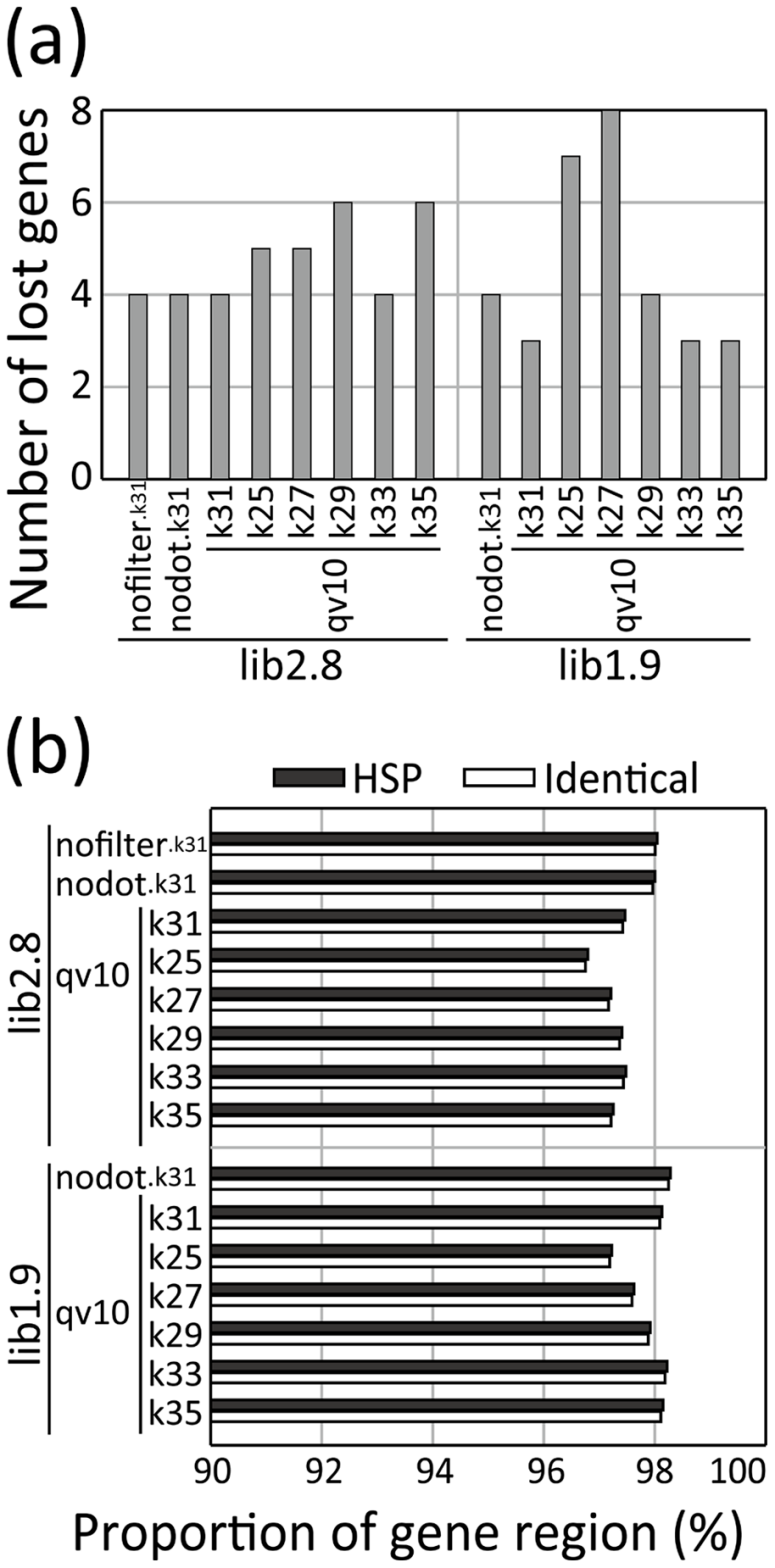
Reconstruction of gene regions in the assembled scaffolds. (a) Plot showing the number of known *Aspergillus oryzae* RIB40 genes that were not found in the assembled genome. (b) Percentages of high-scoring segment pair (HSP; solid) and identical bases (open) in the gene regions. The graph includes the results of the assemblies using lib2.8 and lib1.9 with either unfiltered (nofilter), no undetermined bases (nodot), or QV >10 data. For lib2.8.qv10 and lib1.9.qv10, the results using k-mers of 25 to 35 are included.

We investigated the degree of reconstruction of gene regions and clusters that is valuable information of fungal genomes. As shown in Fig. 3, our *de novo* assembly process was able to reconstruct most reference gene sequences with good accuracy. In the assembled scaffolds of lib2.8.nofilter.k31, only 4 (∼0.3%) of 12,064 genes in the reference genome were completely lost (Fig. 3a). Based on the Blastn analysis, the assembled sequences covered 98% of the reference gene nucleotide sequences by HSPs, with almost all of these sequences being completely identical (Fig. 3b).

The most representative SMB proteins are PKS and NRPS, whose encoding genes contained conserved sequence motifs [34]. Because PKS and NRPS genes consist of similar repeating units, it is difficult to correctly assemble these gene sequences using only short reads. Here, in lib2.8.nofilter.k31, 96.78% of the PKS and NRPS gene sequences formed HSP with the assembled scaffolds (Table 3). Although the value is ∼1.2% smaller than that of all genes, we demonstrated that a set of SOLiD short sequence reads (50-bp lengths) could highly reconstruct the PKS and NRPS genes containing many similar repeating units.

**Table 3:** Reconstruction of the PKS/NRPS genes and gene clusters.

		Gene continuity in cluster		
Assembly	HSP in PKS/NRPS genes (%)^a^	AO090026000008− AO090026000036 (29 genes, ∼73 kb)	AO090001000018− AO090001000055 (37 genes, ∼75 kb)	AO090113000136− AO090113000138 (3 genes, ∼6 kb)
lib2.8.nofilter.k31	96.78 (98.04)	○	○	○
lib2.8.nodot.k31	95.63 (98.00)	○	○	○
lib2.8.qv10.k31	96.33 (97.47)	○	○	○
lib2.8.qv10.k25	91.73 (96.79)	○	○	○
lib2.8.qv10.k27	92.53 (97.21)	○	○	○
lib2.8.qv10.k29	95.21 (97.41)	○	○	○
lib2.8.qv10.k33	95.30 (97.48)	○	○	○
lib2.8.qv10.k35	94.73 (97.24)	○	○	○
lib1.9.nodot.k31	97.88 (98.28)	○	○	○
lib1.9.qv10.k31	96.83 (98.13)	○	○	○
lib1.9.qv10.k25	93.55 (97.22)	○	○	○
lib1.9.qv10.k27	95.00 (97.62)	○	○	○
lib1.9.qv10.k29	96.43 (97.92)	○	○	○
lib1.9.qv10.k33	96.08 (98.22)	○	○	○
lib1.9.qv10.k35	95.50 (98.15)	○	○	○

HSP, high-scoring segment pair; NRPS, nonribosomal peptide synthetase; PKS, polyketide synthase.

The open circles denote that the gene continuity of genes in each corresponding cluster is reproduced.

a The PKS/NRPS genes are listed in Table S2. The values in parentheses are the proportion of HSPs in all genes.

**Figure 4:**
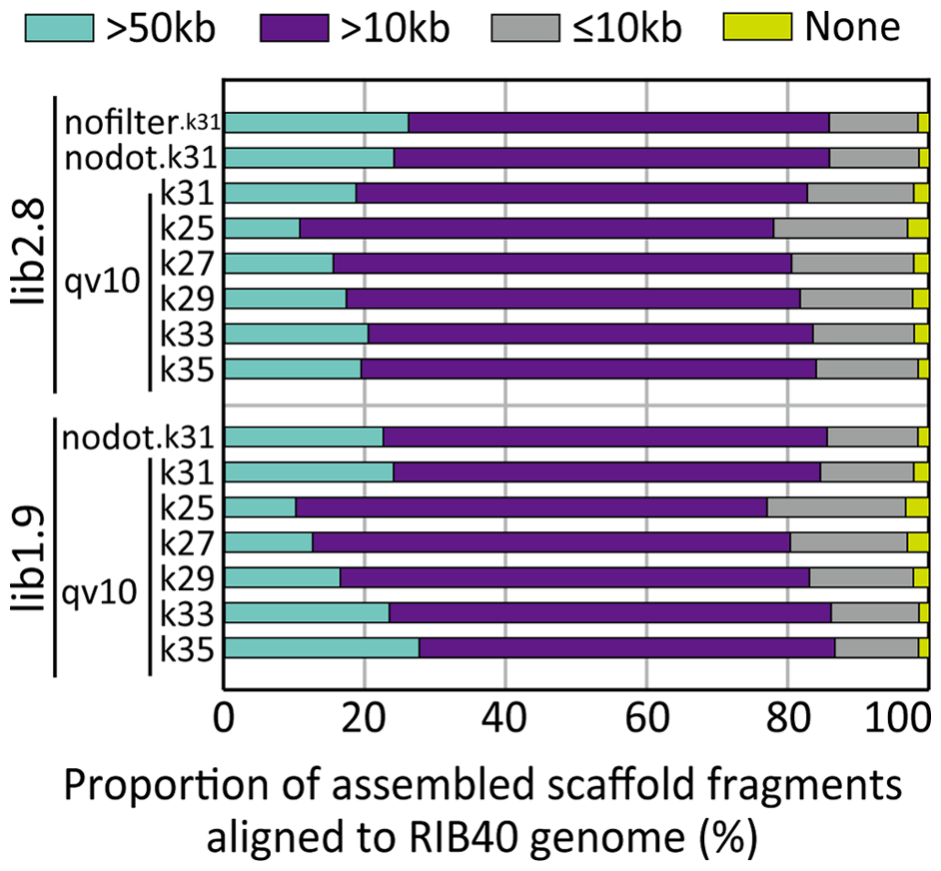
Proportion of assembled scaffold fragments aligned to the *Aspergillus oryzae* RIB40 reference genome. The length of aligned fragments are indicated by color (bluegreen, >50 kb; purple, >10 kb; gray, ≤10 kb; and yellow, 0 or none). The graph includes the results of the assemblies using lib2.8 and lib1.9 with either unfiltered (nofilter), no undetermined bases (nodot), or QV >10 data. For lib2.8.qv10 and lib1.9.qv10, the results using k-mers of 25 to 35 are included.

Genes must be correctly continuous to identify SMB gene clusters. As shown in Figure 4, the assembled scaffolds composed of >50- and >10-kb fragments covered ∼27% and ∼85% of the reference genome, respectively, in lib2.8.nofilter.k31. We also examined gene continuity in the assembled scaffolds using three gene clusters, AO090026000008−AO090026000036 (29 genes, ∼73 kb), AO090001000018−AO090001000055 (38 genes, ∼75 kb), and AO090113000136−AO090113000138 (3 genes, ∼6 kb), which correspond to SMB gene clusters of aflatoxin (*A. flavus*), gliotoxin (*A. flavus*, hypothetical), and KA (*A. oryzae*) [22]. *A. oryzae* does not produce aflatoxin due to several gene deficiencies and mutations [35]–[38], but does have a genomic region corresponding to the aflatoxin biosynthetic gene cluster of *A. flavus* [21], [39]–[41]. Similarly, *A. oryzae* is not reported to produce gliotoxin, but a region with homology to a gliotoxin biosynthetic gene cluster from *A. flavus* was identified in a homology search. As summarized in Table 3, complete gene continuity was preserved in lib2.8.nofilter.k31 and other assemblies. These results suggest that our *de novo* assembly approach can reconstruct SMB gene clusters in a fungal genome sequence.

### Effect of data filtering on assembly performance

SOLiD short reads have a high sequence accuracy of >99.99%; however, reads containing undetermined or low-quality bases are also included. Therefore, excluding low-quality read data is expected to improve assembly accuracy. On the other hand, loss of reads by filtering may lead to inefficient connection of sequence nodes in the de Bruijin graph assembly and scaffolding contigs. To examine the effect of filtering low-quality reads on the assembly performance, we prepared three and two types of read sets for lib2.8 and lib1.9, respectively, in which reads were either unfiltered (nofilter), lacked undetermined bases (nodot), or had bases of QVs >10 (qv10), and executed the assemblies.

**Figure 5:**
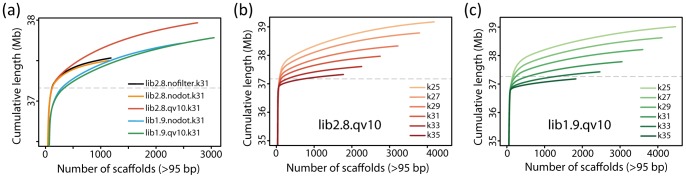
Cumulative lengths of assembled scaffolds (>95 bp). (a) The profiles in the lib2.8.nofilter.k31, lib2.8.nodot.k31, lib2.8.qv10.k31, lib1.9.nodot.k31, and lib1.9.qv10.k31 assemblies using lib2.8 and lib1.9 with either unfiltered (nofilter), no undetermined bases (nodot), or QV >10 data. (b) The profiles of lib2.8.qv10 and (c) lib1.9.qv10 with changing k-mers from 25 to 35. The dashed grey line at 37.2 Mb in each graph denotes the size of the reference genome.

First, we compared the assembly results between lib2.8.nofilter.k31 and lib2.8.nodot.k31. The lib2.8.nofilter.k31 and lib2.8.nodot.k31 assemblies displayed nearly the same pattern of assembled scaffold cumulative lengths; reaching a plateau at ∼37.5 Mb with ∼1000 scaffolds (Fig. 5a). The number and maximum size of assembled scaffolds (Table 2), in addition to the reconstruction percentage of the total genes and genome sequence (Fig. 3 and 4), were also similar for the two assemblies. Therefore, although the N50 value (1.36 Mb) of lib2.8.nodot.k31 was lower than that (1.68 Mb) of lib2.8.nofilter.k31, the overall assembly performance was not significantly affected by unfiltering reads including undetermined bases. The SOLiD *De Novo* Accessory Tools can handle reads including undetermined bases, but it is considered better to exclude such reads because undetermined ‘N’ bases are automatically converted to ‘A’ by the Velvet program.

We next tested the higher degree of data filtering by comparing the assembly results between lib2.8.nodot.k31 and lib2.8.qv10.k31, and lib1.9.nodot.k31 and lib1.9.qv10.k31, and found that filtering low-quality sequence reads reduced misarrangement of the genome sequence. As summarized in Table 4, the numbers of misjoins, inversions, deletions, and insertions (>500 bp), in addition to the total size of the deletions and insertions, were decreased by the data filtering both in lib2.8 and lib1.9.

**Table 4:** Numbers of misjoin, inversion, deletion, and insertion, and total sizes of deletion and insertion (>500 bp).

	Number				Total Size (kb)	
Assembly	Misjoin	Inversion	Deletion	Insertion	Deletion	Insertion
lib2.8.nofilter.k31	798	14	117	91	371	278
lib2.8.nodot.k31	802	12	119	97	379	281
lib2.8.qv10.k31	657	8	101	59	365	143
lib2.8.qv10.k25	729	8	83	67	287	139
lib2.8.qv10.k27	750	9	78	75	271	222
lib2.8.qv10.k29	694	6	132	67	520	182
lib2.8.qv10.k33	713	10	80	81	235	268
lib2.8.qv10.k35	808	10	98	108	248	265
lib1.9.nodot.k31	593	21	121	66	359	153
lib1.9.qv10.k31	498	11	107	37	299	86
lib1.9.qv10.k25	681	16	61	75	196	195
lib1.9.qv10.k27	673	19	109	91	333	185
lib1.9.qv10.k29	550	10	118	53	397	96
lib1.9.qv10.k33	501	13	117	57	332	130
lib1.9.qv10.k35	537	24	126	66	497	134

On the other hand, more scattered scaffolds were generated by the data filtering, as observed for smaller N50 values and larger numbers of scaffolds (Table 2). Redundant scaffolds were also more frequently generated by the data filtering; the cumulative lengths in lib2.8.qv10.k31 (∼38.0 Mb) and lib1.9.qv10.k31 (∼37.8 Mb) were more redundant than that in lib2.8.nodot.k31 (∼37.5 Mb) and lib1.9.nodot.k31 (∼37.7 Mb), respectively, compared to the total size of reference sequence (37.2 Mb, Fig. 5a). In addition, small insertions and deletions in the aligned scaffold fragments were increased by the data filtering in both lib2.8 and lib1.9 (Table 5), denoting that the sequence accuracy was decreased by the data filtering. Decreased sequence accuracy is due to the scaffolding became difficult after filtering sequence reads that hold information for connection of sequence nodes. In fact, as summarized in Table 6, the scaffolding ratio or the ratio of contig number to scaffold number was lowered by the data filtering (lib2.8.qv10.k31, 2.82; lib2.8.nodot.k31, 4.59; lib1.9.qv10.k31, 2.71; and lib1.9.nodot.k31, 2.91).

**Table 5:** Numbers of nucleotide insertions and deletions in assembled sequences aligned to the reference sequence.

	Genome		Gene	
Assembly	Insertion	Deletion	Insertion	Deletion
lib2.8.nofilter.k31	53349	44561	12981	3631
lib2.8.nodot.k31	52701	44467	12634	3540
lib2.8.qv10.k31	59565	44642	17462	4402
lib2.8.qv10.k25	52868	44401	16759	3938
lib2.8.qv10.k27	56416	44551	18245	3704
lib2.8.qv10.k29	57850	45344	17829	4045
lib2.8.qv10.k33	58537	45304	15893	4641
lib2.8.qv10.k35	58438	42832	14916	4082
lib1.9.nodot.k31	57742	40999	16351	2878
lib1.9.qv10.k31	61770	42601	18321	3048
lib1.9.qv10.k25	52683	41800	16941	2621
lib1.9.qv10.k27	56573	41604	18012	2612
lib1.9.qv10.k29	61690	42336	18781	3118
lib1.9.qv10.k33	61623	42193	17735	3384
lib1.9.qv10.k35	62251	42729	16086	3990

**Table 6:** Numbers and ratios of nucleotide contigs and scaffolds generated in each assembly.

Assembly	Contig	Scaffold	Contig/scaffold
lib2.8.nofilter.k31	5332	1184	4.50
lib2.8.nodot.k31	5265	1147	4.59
lib2.8.qv10.k31	7763	2751	2.82
lib2.8.qv10.k25	9448	4182	2.26
lib2.8.qv10.k27	8960	3790	2.36
lib2.8.qv10.k29	8321	3220	2.58
lib2.8.qv10.k33	7179	2260	3.18
lib2.8.qv10.k35	6702	1774	3.78
lib1.9.nodot.k31	7153	2456	2.91
lib1.9.qv10.k31	8254	3050	2.71
lib1.9.qv10.k25	10278	4468	2.30
lib1.9.qv10.k27	9704	4109	2.36
lib1.9.qv10.k29	9066	3601	2.52
lib1.9.qv10.k33	7545	2465	3.06
lib1.9.qv10.k35	6893	1828	3.77

The above two conflicting effects of data filtering delivered different results between lib2.8 and lib1.9. We defined the “real” N50 for reference genome sequences covered by highly accurate sequence fragments of assembled scaffolds (R50), and evaluated the values for each assembly. The R50 value was larger in lib1.9.qv10.k31 (30 kb) than in lib1.9.nodot.k31 (28 kb), whereas it was shorter in lib2.8.qv10.k31 (27 kb) than in lib2.8.nodot.k31 (30 kb, Table 2). As expected, the recovery of gene and genome sequence regions decreased in lib2.8.qv10.k31 from lib2.8.nodot.k31, whereas it almost identical between lib1.9.nodot.k31 and lib1.9.qv10.k31 (Fig. 3, 4). The percentage of scaffold fragments of >50 kb became even larger in lib1.9.qv10.k31 than in lib1.9.nodot.k31 (Fig. 4).

### Effect of library insert size on assembly performance

When using a mate-paired library, the sequence information from such a library is expected to facilitate the assembly of genomic regions with a high repeat content that is smaller than the insert size of the library. On the other hand, if the insert size of a mate-paired library is too large, it may become difficult to scaffold contigs that are smaller than the insert size and are not sufficiently overlapped with each other. To investigate the dependence of *de novo* genome assembly in strain RIB40 on library insert size, we compared the assembly results from the mate-paired libraries lib2.8 and lib1.9, which have different insert sizes of 1.9 and 2.8 kb, respectively. It should be noted that the depth of coverage was lower in the lib1.9 read sets (lib1.9.nodot.k31, 148; lib1.9.qv10.k31, 117) than in those of lib2.8 (lib2.8.nodot.k31, 334; lib2.8.qv10.k31, 228, Table 2), while the read data of lib2.8 and lib1.9 had similar QVs before and after the data filtering (Table S3).

Although the N50 value was smaller (Table 2), the numbers of misjoin and insertion (>500 bp) were decreased in lib1.9.nodot/qv10.k31 compared to lib2.8.nodot/qv10.k31 (Table 4). As a result, the R50 value was comparative between lib1.9 and lib2.8, and was even larger in lib1.9.qv10.k31 than in lib2.8.qv10.k31 (Table 2), even though shorter and more scaffolds were generated in the lib1.9 assemblies. The HSP percentage in gene regions is in accordance with this result (Fig. 3). Thus, although a mate-paired library of shorter insert size may degrade sizes of assembled scaffolds, it appears superior for the reconstruction of genome sequences. With respect to sequence accuracy, the number of deletion errors in scaffold fragments aligned to the reference genome was decreased in lib1.9 compared to lib2.8, although the number of insertions was increased (Table 5).

### Effect of k-mer size on assembly performance

The size of k-mer, the principle parameter for the de Bruijn graph algorithm in Velvet, is known to affect assembly performance. For example, Haridas et al. [12] reported that Velvet yielded the largest N50 value when using a k-mer of 43 for Illumina paired-end reads with a length of 75 bp. In the algorithm, sequence nodes having bases of k-mer size are created from read data and are then connected to yield as many nodes as possible in a path or sequence [25]. Therefore, k-mers of adequately large size are expected to increase assembly performance. To estimate the most adequate k-mer size, we performed *de novo* genome assemblies with changing k-mer size from 25 to 35 (restricted to odd integers) for the read sets of lib2.8.qv10 and lib1.9.qv10, and compared the results.

**Figure 6:**
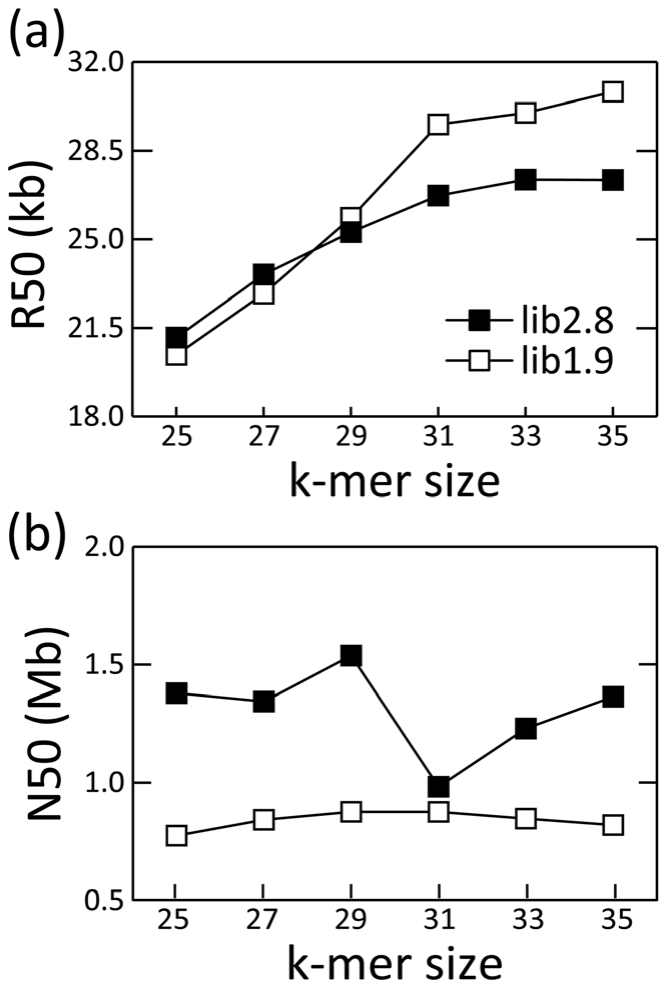
R50 and N50 values with different k-mer sizes. (a) The R50 and (b) the N50 values for lib2.8.qv10 (closed square) and lib1.9.qv10 (open square). The R50 value corresponds to N50 using sequence fragments of the reference genome covered by highly accurate sequences of assembled scaffolds.

The profile of cumulative lengths shows that less redundant scaffolds were generated when using larger k-mer size in both lib2.8.qv10 and lib1.9.qv10 (Fig. 5b, c). The scaffolding ratio, the ratio of contig number to scaffold number, also increased using the larger k-mer size (Table 6). As a result, the reference gene and genome sequences were well reconstructed with longer scaffold fragments when using a longer k-mer size up to 33 (Fig. 3b, 4). The R50 value increased with larger k-mer sizes, and reached a plateau at k-mer of 33 in lib2.8 (Fig. 6a). The k-mer size improved the assembly results more in lib1.9 than in lib2.8; the R50 values were slightly smaller in lib1.9 than in lib2.8 when the k-mer size was under 29, but became longer in lib1.9 when k-mer was 29 or larger. It should be noticed that the profile of N50 is different from that of R50 because N50 does not include the effect of misarrangements. If using N50 as a criteria, the most adequate k-mer size would be 29 in both lib2.8.qv10 and lib1.9.qv10 (Fig. 6b), but this is not sufficient for correctly estimating assembly performance.

A positive effect of using a smaller k-mer size is that it reduced the number of insertions in the scaffold fragments aligned to the assembled sequences, whereas that of deletions was dependent on a library rather than k-mer size (Table 5).

## Discussion

To our knowledge, this is the first successful report of *de novo* sequencing of a fungal genome using only SOLiD short sequence reads. Our assembled RIB40 genome had a N50 of 1.7 Mb, which is more than 22 times longer than the longest result of 76.9 kb for a fungus using only the SOLiD platform [13]. More than 98% of the bases in the gene regions were reconstructed, and more than 85% and 25% of the reference sequence was covered by assembled scaffold fragments with lengths of >10 and >50 kb, respectively. The assemblies were also able to reconstruct ∼97% of the sequences of the representative secondary metabolite biosynthetic genes, PKS and NRPS, despite the fact that both genes contain numerous repeating units. Based on these findings in *A. oryzae* RIB40, we conclude that the *de novo* sequencing of fungal genomes using only SOLiD short reads is practical and feasible for the detection of fungal genes and SMB gene clusters, when a mate-paired library with an insert size of ∼2 kb and a depth of coverage of ∼×150 are used. Considering that successful *de novo* assembly with Illumina data was achieved with various MP libraries of different insert sizes [14], [15], our *de novo* assembly has still great advantage because it requires only a single SOLiD mate-paired library having an appropriate insert size.

We also investigated the effect of data filtering, library insert size, and k-mer size on assembly performance. The summary of the results and our recommendation drawn from this study are presented in Table 7. A tendency for a trade-off between scaffold size distribution and genome reconstruction was observed except when changing k-mer sizes. Although both data filtering and use of a mate-paired library with shorter insert size decreased the N50 value or generated shorter and more scaffolds, they yielded fewer misarrangements in the assembled scaffolds. As a result, the “real” N50 for scaffold fragments aligned to the reference genome (R50) was comparative in between lib1.9.qv10.k31 and lib2.8.nodot.k31. Thus, we conclude that data filtering can improve genome reconstruction, if the N50 value is not significantly degraded. We recommend assembly of at least two or more data sets having different degree of filtering and choosing the assembly result from the most filtered data unless the N50 is significantly degraded. Longer k-mer sizes improved the N50 (up to 29) and R50 (up to 33), thus a k-mer size of 33 is recommended when the short read length is 50 bp. Sequence accuracy of the scaffold fragment aligned to the reference sequences was decreased by either data filtering or using longer k-mer.

**Table 7:** Summary of our *de novo* genome assembly performance.

	Data filtering	Smaller insert size of a mate-paired library	Longer k-mer size (>25)
Scaffold size distribution (N50)	Decreased	Decreased	Increased (up to ∼29)
Genome reconstruction (R50)	Increased in lib1.9; decreased in lib2.8	Increased (after data filtering)	Increased (up to ∼33)
Sequence accuracy	Degraded	Improved in insertions; degraded in deletions	Degraded
Recommended condition	Filtering reads including undefined (dot) bases	∼2.0 kb	33 (when the read length is 50)

The open circle and cross denote improvement and degradation, respectively, for each entry of the assembly performance.

Degraded in lib2.8 but improved in lib1.9.

Maximum at k-mer size of 29 in both lib2.8 and lib1.9.

The data filtering generated shorter and more scaffolds. As Lin et al. [42] reported, a depth of coverage over 40 does not affect the N50 length of scaffolds assembled by Velvet. Therefore, our findings may not be due to the lowered depth of coverage throughout the reference genome sequence, but were likely due to an excess of deficient reads in specific regions of the RIB40 genome as a result of the data filtering. This is supported by observing the coverage of reference sequence by the raw short reads decreased by 0.01% (∼5 kb) after the data filtering involving qv10 for both lib2.8 and lib1.9 (Table S3). In regard to the insert size of a mate-paired library, the lib1.9 read sets with 1.9-kb insert size showed smaller N50 values than the lib2.8 ones with 2.8-kb insert size, but showed better reconstruction of the reference genome sequence and gene regions. A mate-paired library having an insert size of ∼3.0 kb can yield long scaffolds, but there is an associated risk decreasing degree of genome reconstruction.

The major NGS platform used for *de novo* genome sequencing is currently that of Illumina. To date, greater than 1000-fold more data sets generated using the HiSeq 2000 system, which is the most recent Illumina platform, have been deposited in public databases, including those of the National Center for Biotechnology Information, European Bioinformatics Institute, and DNA Data Bank of Japan (as of 7/5/2012), than those from the SOLiD 5500 system, the latest SOLiD platform. Presently, SOLiD short reads are not generally recognized as being suitable for *de novo* genome sequencing; however, our present results provide counter evidence to this idea. Although we used the SOLiD 5500xl system in this study, we confirmed that read data from the SOLiD 3 Plus platform also exhibited assembly performance that was comparable to that of the 5500×l (data not shown).

Our assembly result with the N50 of 1.7 Mb is considered to be long sufficient for fungal genomes, which consist of several chromosomes and having AT-rich centromere sequences that is difficult to be read. In the *A. oryzae* genome, the longest chromosome has the size of ∼6.5 Mb, but the sequence of the chromosome would be divided into two or more scaffolds by the AT-rich centromere; thus the longest scaffold would have the size of ∼3 Mb at most. Even though our assembly yielded sufficiently long scaffolds, there will still remain a room for improvement. Gnerre et al. reported the algorithm ALLPATHS-LG that can yield a N50 of 11.5 Mb for human genome using only the Illumina platform, by several improvements including handling of repetitive sequences and low coverage regions from the previous version of their algorithm, ALLPATHS 2 [14], [43]. Since the longest N50 for fungi by ALLPATHS 2 was 222 kb, our pipeline may also be able to generate more long and accurate sequences by similar improvements.

The assembly pipeline can be executed on an average desktop computer, with the following recommended specifications; Intel Core i7-3930K CPU (3.2 GHz, six cores), 64 GB memory (DDR3 PC3-10600 DIM 8GB×8), and 2 TB storage. Among these recommended specifications, memory size is the most critical for the smooth running of the assembly; 64 GB or more is required for the *de novo* assembly of a fungal genome of 40 Mb. In addition, the assemblies can be performed on Linux operating systems, such as CentOS and Ubuntu, which are available for free. Therefore, *de novo* genome assembly can be performed in any laboratory if short read sequence data is obtained, with a possibility that will expand the boundaries of fungal studies.

### Conclusions

*De novo* genome assembly using only SOLiD short reads is practical and feasible for fungal genomes if mate-paired libraries with ∼2-kb insert sizes, read data with a depth of coverage of ∼100 fold, and k-mer size of ∼33 (when the read length is 50 bp) are used. Using this approach, we reconstructed >98% of the gene regions in the *A. oryzae* RIB40 genome with an N50 of 1.7 Mb. We also demonstrated that mild data filtering, such as excluding reads with undetermined bases, yielded long and accurate scaffolds. Taken together, our findings suggest that accurate and high-throughput SOLiD platforms that generate short reads of ∼60 bp can be utilized for the *de novo* sequencing of fungal genomes. This approach may be improved when using data from developing NGS technologies that yield long sequences (>1 kb) with low accuracy in combination.

## Supporting Information

Table S1 **Parameters used in the de novo genome assemblies.**
(DOC)

Table S2 **PKS and NRPS genes in** ***A. oryzae*** **RIB40.**
(DOC)

Table S3 **Quality of read data generated from libraries lib2.8 and lib1.9.**
(DOC)
