# Supplementary material for: Fine De Novo Sequencing of a Fungal Genome Using only SOLiD Short Read Data: Verification on Aspergillus oryzae RIB40
Source: PLoS One. 2013 May 7;8(5):e63673. doi: 10.1371/journal.pone.0063673 (PMC3646829; doi:10.1371/journal.pone.0063673)
Supplement: Table S1 — Parameters used in the de novo genome assemblies. (DOC) [file pone.0063673.s001.doc]

# Table S1. Parameters used in the *de novo* genome assemblies.

|  | Assembly | ins_length | ins_length_sd | min_contig_lgth | min_pair_count | exp_cov | cov_cutoff | read_trkg |  |
| --- | --- | --- | --- | --- | --- | --- | --- | --- | --- |
|  | lib2.8.nofilter.k31 | 2764 | 500 | 100 | 136 | 169 | 13 | yes |  |
|  | lib2.8.nodot.k31 | 2764 | 500 | 100 | 134 | 167 | 13 | yes |  |
|  | lib2.8.qv10.k31/k25/k27/k29/k33/k35 | 2764 | 500 | 100 | 92 | 114 | 9 | yes |  |
|  | lib1.9.nodot.k31 | 1875 | 400 | 100 | 60 | 74 | 7 | yes |  |
|  | lib1.9.qv10.k31/k25/k27/k29/k33/k35 | 1875 | 400 | 100 | 47 | 59 | 6 | yes |  |
