# Supplementary material for: Fine De Novo Sequencing of a Fungal Genome Using only SOLiD Short Read Data: Verification on Aspergillus oryzae RIB40
Source: PLoS One. 2013 May 7;8(5):e63673. doi: 10.1371/journal.pone.0063673 (PMC3646829; doi:10.1371/journal.pone.0063673)
Supplement: Table S2 — PKS and NRPS genes in A. oryzae RIB40. (DOC) [file pone.0063673.s002.doc]

**Table S2.** PKS and NRPS genes in *A. oryzae* RIB40^*^

| PKS | Length | NRPS | Length |
| --- | --- | --- | --- |
| AO090001000277 | 11841 | AO090001000009 | 9504 |
| AO090001000293 | 7521 | AO090001000043 | 4927 |
| AO090001000402 | 7427 | AO090001000262 | 16343 |
| AO090001000506 | 8583 | AO090001000516 | 3306 |
| AO090005000798 | 8187 | AO090003000945 | 3583 |
| AO090005000961 | 6545 | AO090003001097 | 4452 |
| AO090009000052 | 7996 | AO090003001545 | 3066 |
| AO090009000071 | 9446 | AO090005000690 | 3837 |
| AO090009000078 | 2673 | AO090005000993 | 5207 |
| AO090009000131 | 8234 | AO090005001026 | 2429 |
| AO090010000048 | 6466 | AO090010000498 | 3227 |
| AO090010000114 | 7791 | AO090011000043 | 20892 |
| AO090010000402 | 7640 | AO090020000240 | 3129 |
| AO090010000404 | 6762 | AO090020000380 | 11964 |
| AO090011000015 | 7656 | AO090023000528 | 14341 |
| AO090020000186 | 8034 | AO090026000378 | 3216 |
| AO090023000444 | 5447 | AO090026000585 | 4017 |
| AO090023000877 | 7003 | AO090038000149 | 2888 |
| AO090026000001 | 1754 | AO090038000390 | 16015 |
| AO090026000009 | 6624 | AO090038000543 | 11325 |
| AO090026000149 | 7483 | AO090038000550 | 3027 |
| AO090038000086 | 8396 | AO090102000338 | 7112 |
| AO090038000210 | 8340 | AO090102000465 | 9036 |
| AO090102000166 | 7290 | AO090103000167 | 5772 |
| AO090102000545 | 6651 | AO090103000223 | 4111 |
| AO090103000224 | 11701 | AO090103000355 | 15692 |
| AO090113000209 | 3793 | AO090120000024 | 5256 |
| AO090206000074 | 5301 |  |  |
| AO090701000530 | 6075 |  |  |
| AO090701000826 | 7890 |  |  |

*A. oryzae*, *Aspergillus oryzae*.

# ^*^ Genes were identified by searching the terms “Polyketide (PKS)” or “Non-ribosomal (NRPS)” in the annotations.
