# Supplementary material for: Fine De Novo Sequencing of a Fungal Genome Using only SOLiD Short Read Data: Verification on Aspergillus oryzae RIB40
Source: PLoS One. 2013 May 7;8(5):e63673. doi: 10.1371/journal.pone.0063673 (PMC3646829; doi:10.1371/journal.pone.0063673)
Supplement: Table S3 — Quality of read data generated from libraries lib2.8 and lib1.9. (DOC) [file pone.0063673.s003.doc]

## Table S3. Quality of read data generated from libraries lib2.8 and lib1.9.

|  | Library | Number of read pairs | Read pairs including bases of no-call (%) | Average QV |  |  | Coverage by reads (%) |  |  |
| --- | --- | --- | --- | --- | --- | --- | --- | --- | --- |
|  |  |  |  | nodot | qv10 |  | nodot | qv10 |  |
|  | lib2.8 | 112347006 | 0.67 | 26.77 | 27.88 |  | 99.96 | 99.95 |  |
|  | lib1.9 | 50526687 | 1.13 | 26.76 | 27.90 |  | 99.91 | 99.90 |  |

QV, quality value.
